# Validity and applicability of the global leadership initiative on malnutrition criteria in non-dialysis patients with chronic kidney disease

**DOI:** 10.3389/fnut.2024.1340153

**Published:** 2024-02-01

**Authors:** Hui Huang, Qian Wang, Yayong Luo, Zhengchun Tang, Fang Liu, Ruimin Zhang, Guangyan Cai, Jing Huang, Li Zhang, Li Zeng, Xueying Cao, Jian Yang, Yong Wang, Keyun Wang, Yaqing Li, Qihu Li, Xiangmei Chen, Zheyi Dong

**Affiliations:** ^1^Department of Nephrology, First Medical Center of Chinese PLA General Hospital, National Key Laboratory of Kidney Diseases, National Clinical Research Center for Kidney Diseases, Beijing Key Laboratory of Kidney Diseases Research, Beijing, China; ^2^School of Clinical Medicine, Guangdong Pharmaceutical University, Guangzhou, China; ^3^Chengdu University of Traditional Chinese Medicine, Chengdu, China

**Keywords:** GLIM, SGA, PEW, malnutrition, chronic kidney disease, validation

## Abstract

**Introduction:**

There are no standardized assessment criteria for selecting nutritional risk screening tools or indicators to assess reduced muscle mass (RMM) in the Global Leadership Initiative on Malnutrition (GLIM) criteria. We aimed to compare the consistency of different GLIM criteria with Subjective Global Assessment (SGA) and protein-energy wasting (PEW).

**Methods:**

In this study, nutritional risk screening 2002 first four questions (NRS-2002-4Q), Nutritional Risk Screening 2002 (NRS-2002), Malnutrition Universal Screening Tool (MUST), and Mini-Nutritional Assessment Short-Form (MNA-SF) tools were used as the first step of nutritional risk screening for the GLIM. The RMM is expressed using different metrics. The SGA and PEW were used to diagnose patients and classify them as malnourished and non-malnourished. Kappa (κ) tests were used to compare the concordance between the SGA, PEW, and GLIM of each combination of screening tools.

**Results:**

A total of 157 patients were included. Patients with Chronic kidney disease (CKD) stage 1–3 accounted for a large proportion (79.0%). The prevalence rates of malnutrition diagnosed using the SGA and PEW were 18.5% and 19.7%, respectively. The prevalence of GLIM-diagnosed malnutrition ranges from 5.1% to 37.6%, depending on the different screening methods for nutritional risk and the different indicators denoting RMM. The SGA was moderately consistent with the PEW (κ = 0.423, *p* < 0.001). The consistency among the GLIM, SGA, and PEW was generally low. Using the NRS-2002-4Q to screen for nutritional risk, GLIM had the best agreement with SGA and PEW when skeletal muscle index (SMI), fat-free mass index (FFMI), and hand grip strength (HGS) indicated a reduction in muscle mass (SGA: κ = 0.464, 95% CI 0.28–0.65; PEW: κ = 0.306, 95% CI 0.12–0.49).

**Conclusion:**

The concordance between the GLIM criteria and the SGA and PEW depended on the screening tool used in the GLIM process. The inclusion of RMM in the GLIM framework is important. The addition of HGS could further improve the performance of the GLIM standard compared to the use of body composition measurements.

## 1 Introduction

Chronic kidney disease (CKD) is one of the diseases recognized worldwide as a serious threat to public health and is characterized by high incidence and poor prognosis. Currently, the global average incidence of CKD is 9.1% (1). The incidence of this disease in the Chinese population is approximately 10.8%, and the number of patients suffering from this disease is as high as 120 million (2).

CKD is characterized by structural and functional changes in the kidneys that last more than 3 months (3). CKD involves multiple systems, including the respiratory, circulatory, skeletal, and endocrine systems, and eventually progresses to end-stage renal disease (ESRD). Although it is possible to prolong the life of patients with ESRD through dialysis and kidney transplantation, this also imposes a significant economic burden on patients. Malnutrition, as a more common complication in CKD patients, is a risk factor for cardiovascular events and death in CKD patients (4). Early recognition of malnutrition and protein-energy wasting (PEW) can shorten the length of hospital stays, reduce hospital costs, and improve quality of life (5, 6).

The 2020 updated National Kidney Foundation’s Kidney Disease Outcomes Quality Initiative (KDOQI) nutritional guidelines (7) recommend standardized CKD Nutritional Screening. The Subjective Global Assessment (SGA), a widely recognized tool for assessing nutritional status in clinical practice which can predict morbidity and mortality associated with malnutrition. The SGA has been validated in the CKD population and is considered the gold standard for nutritional status assessment (8–10). In 2008, the International Society of Renal Nutrition and Metabolism (ISRNM) proposed the PEW assessment, which describes catabolic disorders in patients with CKD due to malnutrition and metabolic disorders (11). PEW is associated with increased morbidity, hospital admissions, and increased risk of infection and death (12).

In 2019, the Global Leadership Initiative on Malnutrition (GLIM) was proposed to establish a global consensus on the core diagnostic criteria for malnutrition in adults in a clinical setting. The diagnosis of malnutrition can be divided into two steps: screening and assessment (13). The first step used a validated nutritional screening tool; however, it is not clear which tool to choose because different nutritional risk screening tools have different characteristics. For example, the Nutritional Risk Screening 2002 (NRS-2002) is a universally accepted screening tool for nutritional risk in hospitalized patients (14). The nutritional risk screening 2002 first four questions (NRS-2002-4Q) can be used independently to screen for nutritional risk (15). The Malnutrition Universal Screening Tool (MUST) is a simple, fast, and commonly used nutritional screening tool. The Mini-Nutritional Assessment Short-Form (MNA-SF) is commonly used to assess the risk of malnutrition in older adults (16, 17). The second step is the assessment and grading of malnutrition severity of malnutrition, which should include at least one phenotypic and one etiological criterion (18). Reduced muscle mass (RMM), as a phenotypic criterion in the GLIM process, has been uniformly assessed (19). Because of the lack of accurate and popular measurement condition, RMM has often been omitted in recent studies (20, 21). The GLIM guidelines suggest that skeletal muscle index (SMI) and fat-free mass index (FFMI) may be used for RMM and that hand grip strength (HGS) is also a supportive measure (19); however, the GLIM is a new nutritional score that has not been validated in patients with non-dialysis CKD. Therefore, we aimed to compare the consistency of the GLIM criteria for the diagnosis of malnutrition in CKD patients with SGA and PEW when different screening tools and the different methods used for the assessment of RMM were applied in the GLIM process.

## 2 Materials and methods

### 2.1 Study population

This cross-sectional study was conducted from March to October 2022 in adults with CKD who were hospitalized at the Department of Nephrology of the First Medical Center of the Chinese People’s Liberation Army General Hospital. All patients with CKD met the following criteria: (1) diagnosis of CKD according to the 2012 KDIGO Clinical Practice Guidelines (3), (2) age ≥ 18 years. The exclusion criteria were as follows: (1) patients with a history of severe infection within the past month; (2) patients with acute and severe diseases in the past 6 months; (3) patients with malignant tumors; (4) pregnant or lactating women; and (5) incomplete medical history or clinical examination results. Finally, 157 non-dialysis CKD patients were included in the analysis. This study was conducted in accordance with the Declaration of Helsinki and was approved by the Ethics Committee of the Chinese People’s Liberation Army General Hospital (approval number S2022-324-01). All participants provided signed informed consent and agreed to participate in the study.

### 2.2 Anthropometry and body composition

Data on the patients’ body mass index (BMI), HGS, and body composition were collected, calculated, and measured. BMI was calculated by dividing weight (in kilograms) by the square of height (in meters). Regarding HGS, the handle of the handgrip dynamometer was adjusted according to the size of the participant’s hand, and the participant was instructed to hold the handgrip dynamometer in a standing position, with the arm hanging down naturally, and to grip the dynamometer as hard as possible with one hand. Measurements were taken to an accuracy of 0.1 kg and repeated three times at 1-min intervals, with the highest value taken as the HGS value.

InBody S10 (InBody, Seoul, South Korea) with a segmental multifrequency approach (1 kHz, 5 kHz, 50 kHz, 250 kHz, 500 kHz, and 1 MHz) was used to estimate body composition, including SMI and fat-free mass (FFM). FFMI was calculated as FFM divided by height squared.

All patients were asked not to consume any food or drink and avoid strenuous activity within 4 h before testing. Generally, this device applies a small alternating current to the body through tetrapolar eight-point tactile electrodes and separately measures the impedance of the arms, legs, and trunk at different frequencies.

### 2.3 The GLIM approach

**Figure 1:**
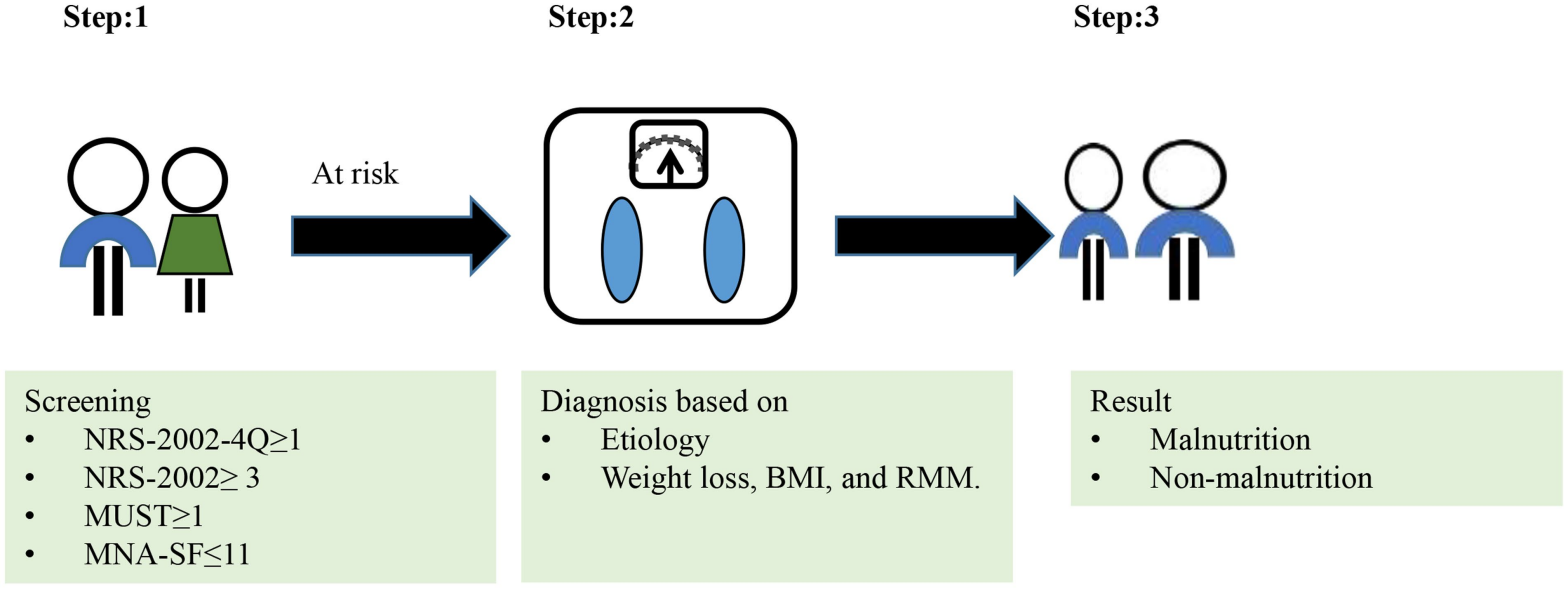
The GLIM approach.

The GLIM approach is shown in Figure 1. In Step 1 of the GLIM approach, we used without risk screening tool and four different tools to screen for risk of malnutrition, such as NRS-2002-4Q, NRS-2002, MUST or MNA-SF. In Step 2 of the GLIM approach, the diagnosis of malnutrition was confirmed.

Phenotypic criteria:

Low body mass index (BMI): Asia: <18.5 kg/m^2^ if <70 years, or < 20 kg/m^2^ if >70 years (18).
Unintentional body weight loss: >5% within past 6 months, or > 10% beyond 6 months (18).

For RMM, we have a different definition.

RMM1: no indicator of RMM.
RMM2: meeting “SMI” or “FFMI.”
RMM3: meeting at least one criterion of “SMI,” “FFMI,” and “HGS.”

The cutoff values for low muscle mass were set to SMI <5.7 kg/m^2^ for women and < 7.0 kg/m^2^ for men (13). Similarly, the cutoff values of FFMI for low muscle mass loss was <15 kg/m^2^ for women and < 17 kg/m^2^ for men (13). The cutoff values for HGS were set at <18 kg for women and < 28 kg for men (22).

Etiologic criteria:

Reduced intake or assimilation: Inadequate protein and energy intake [protein < 0.6 g/kg/d or energy < 25 kcal/kg/d through the 24-h dietary review (7)] or reduced past-week intake.
Disease burden or inflammation: Inflammation is identified by serum albumin < 3.5 g/dL (23) or neutrophil-to-lymphocyte ratio (NLR; ≥3 or <0.7) (24), or plasma C-reactive protein (CRP) > 8 mg/L (25).

As for GLIM, different definitions were used for the diagnosis of malnutrition.

GLIM1: Phenotypic criteria (low BMI, unintentional body weight loss or RMM1) and Etiologic criteria.
GLIM2:Phenotypic criteria (low BMI, unintentional body weight loss or RMM2) and Etiologic criteria.
GLIM3:Phenotypic criteria (low BMI, unintentional body weight loss or RMM3) and Etiologic criteria.

To diagnosis malnutrition, at least one phenotypic criterion and at least one etiologic criterion should be met.

#### 2.3.1 NRS-2002-4Q

The NRS-2002-4Q is the first step in the initial nutritional screening of the NRS-2002 and consists of four questions. The NRS-2002-4Q independently predicts nutritional risk and is a strong predictor of morbidity and mortality in hospitalized patients (15). In a cross-sectional study of 426 patients with rectal cancer, the NRS-2002-4Q was a more appropriate nutritional screening tool, with a kappa (κ) value of 0.49, sensitivity of 63%, and PPV of 53% (26). For the purposes of this study, NRS-2002-4Q ≥ 1 (out of 4) was defined as malnutrition risk.

#### 2.3.2 NRS-2002

The NRS-2002 was developed by an expert panel from the European Society for Parenteral and Enteral Nutrition (ESPEN) and is a core indicator of nutritional risk based on 128 randomized controlled trials (14). NRS-2002 scores range from 0 to 7, with NRS-2002 ≥ 3 indicating that the patient is at nutritional risk.

#### 2.3.3 MUST

The MUST is a simple and rapid nutritional screening tool (3–5 min) developed by The British Association for Parenteral and Enteral Nutrition (BAPEN) (27, 28). A MUST score of ≥1 out of 3 was used to define malnutrition risk.

#### 2.3.4 MNA-SF

The Mini-Nutritional Assessment Short-Form (MNA) was developed as a malnutrition screening tool for elderly hospitalized patients (29). The MNA-SF has good specificity and sensitivity for the diagnosis of malnutrition in the elderly population (30, 31). An MNA-SF score of ≤11 out of 14 was used to define malnutrition risk.

### 2.4 The SGA

The SGA is a reliable and valid nutritional assessment tool for the diagnosis of malnutrition. It is based on medical history and physical examination, including five history components of weight change, change in food intake, gastrointestinal symptoms, change in mobility, and metabolic demands of the disease state; and three physical examination components of loss of subcutaneous fat, muscle atrophy, and edema (32). Patients with a rating of A were characterized as well-nourished, and those with a rating of B or C were characterized as malnourished.

### 2.5 The PEW

PEW was diagnosed based on the 2008 diagnostic criteria of the ISRNM, with at least three of four criteria: biochemistry, body mass, muscle mass, and dietary intake (11). Dietary intake was derived from a 24-h dietary review, and protein and energy were calculated according to the Dietary Reference Intakes for Chinese People (33).

### 2.6 Statistical analysis

Count data are expressed as frequency (percentage), and comparisons between the two groups were made using the chi-square test or Fisher’s exact test. Sensitivity, specificity, positive predictive value (PPV), and negative predictive value (NPV) were calculated for each screening tool combination using the SGA as the reference method. Agreement between SGA, PEW, and GLIM was tested using κvalues (0–0.2 for poor consistency, 0.21–0.4 for general consistency, 0.41–0.6 for moderate consistency, 0.61–0.8 for good consistency, >0.8 for almost complete consistency). A sensitivity, specificity, PPV, and NPV of 80% for the diagnosis of malnutrition were interpreted as acceptable, as suggested by the GLIM criteria (34). Statistical analyses were performed using SPSS version 26.0 for Mac software (SPSS Inc., Chicago, IL). Statistical significance was set at *p* < 0.05.

### 2.7 Sample size calculation

Post-hoc error size analysis for sensitivity and specificity showed that, using a sensitivity and specificity of 68.8% and a malnutrition rate of 37.6% (corresponding to the actual results in the present study), 95% confident that the value of sensitivity obtained from our sample was within 10 percentage points of the true value, and the specificity was within a distance of 10 percentage points from the true value (35).

## 3 Results

### 3.1 Participant screening and CKD staging

**Figure 2:**
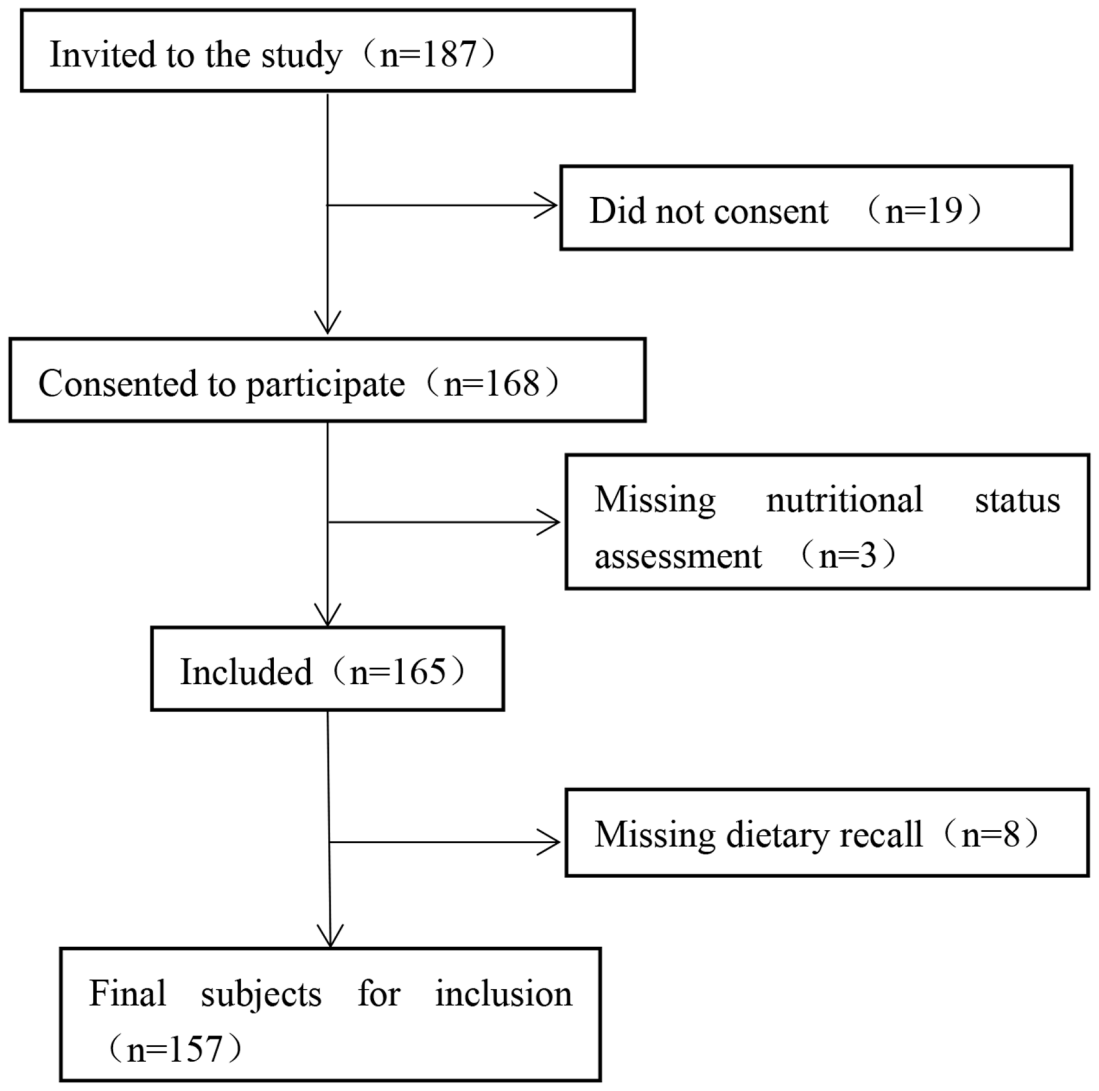
Flow chart of participant screening.

A total of 187 patients with CKD hospitalized in the Department of Nephrology of the First Medical Center of the Chinese People’s Liberation Army General Hospital between March and October 2022 were selected. After excluding 19 patients who did not agree to participate, 3 patients without nutritional status assessment, and 8 patients with missing dietary recall, 157 participants were eligible for inclusion. The participant screening process is illustrated in Figure 2.

Among the 157 patients with CKD, 46 (29.3%) had CKD1, 33 (21.0%) had CKD2, 45 (28.7%) had CKD3, 16 (10.2%) had CKD4, and 17 (10.8%) had CKD5. Patients with CKD stage 1–3 constituted a large proportion of the study population. The characteristics of the 157 eligible patients are shown in Table 1. Of them, 97 (61.7%) were male, 73 (46.4%) had type 2 diabetes mellitus, and 119 (75.7%) had hypertension.

**Table 1:** Baseline characteristics of the study population.

Variable	All participants (*n* = 157)
Age, median (25th–75th percentile)	52 (39.5–61.5)
**Gender, *n* (%)**	
Men	97 (61.7%)
Women	60 (38.3%)
**Anthropometric measures**	
Weight, kg, median (25th–75th percentile)	69 (62.0–79.5)
BMI, kg/m^2^, mean (SD)	25 (3.6)
HGS, kg, mean (SD)	29 (10.0)
**Body composition (BIA)**	
SMI, kg/m^2^, mean (SD)	9 (1.4)
Fat-free mass, kg, mean (SD)	54 (10.6)
Fat-free mass index, kg/m^2^, mean (SD)	19 (2.6)
**Results of laboratory tests**	
Serum creatinine (umol/L), median (25th–75th percentile)	109.2 (74.75–176.20)
eGFR (mL/min/1.73 m^2^), median (25th–75th percentile)	61.1 (32.50–93.54)
Total protein (g/L), mean (SD)	57.3 (9.73)
Serum albumin (g/L), median (25th–75th percentile)	36.6 (30.35–40.25)
Fasting glucose mmol/L, median (25th–75th percentile)	4.7 (4.15–5.32)

BMI, body mass index; SD, standard deviation; SMI, skeletal muscle index; HGS, handgrip strength; T2DM, Type 2 diabetes mellitus; eGFR, estimated Glomerular Filtration Rate.

### 3.2 Nutritional status

As shown in Supplementary Table 1, the prevalence of malnutrition diagnosed according to the SGA and PEW was 18.5% (*n* = 29) and 19.7% (*n* = 31), respectively, with no statistically significant difference between sexes (*p* > 0.05). In the SGA, all participants (100%) had low metabolic demand stress, 40.8% (*n* = 64) had decreased mobility, 36.9% (*n* = 58) had subcutaneous fat loss, and 22.9% (*n* = 36) had edema. Muscle atrophy was observed in 26 patients (16.6%), decreased dietary intake in 12.1% (*n* = 19), gastrointestinal symptoms in 7.6% (*n* = 12), and weight loss in 7% (*n* = 11). None of the 8 items assessed by the SGA were statistically different between the sexes.

In the PEW group, the majority (*n* = 116, 73.9%) of the patients had inadequate dietary protein or energy intake. A total of 58.6% (*n* = 92) of patients had below-normal biochemistry. 53 patients (33.8%) had the following body mass characteristics: BMI < 23 kg/m^2^, weight loss >5% in 3 months, weight loss of 6 months >10%, or body fat percentage (BFP) < 10%. 16 patients (10.2%) had muscle mass loss. In the gender intergroup comparison, the proportion of patients with body mass loss was significantly higher in females than in males (46.7% vs. 25.8%; *p* = 0.007).

**Figure 3:**
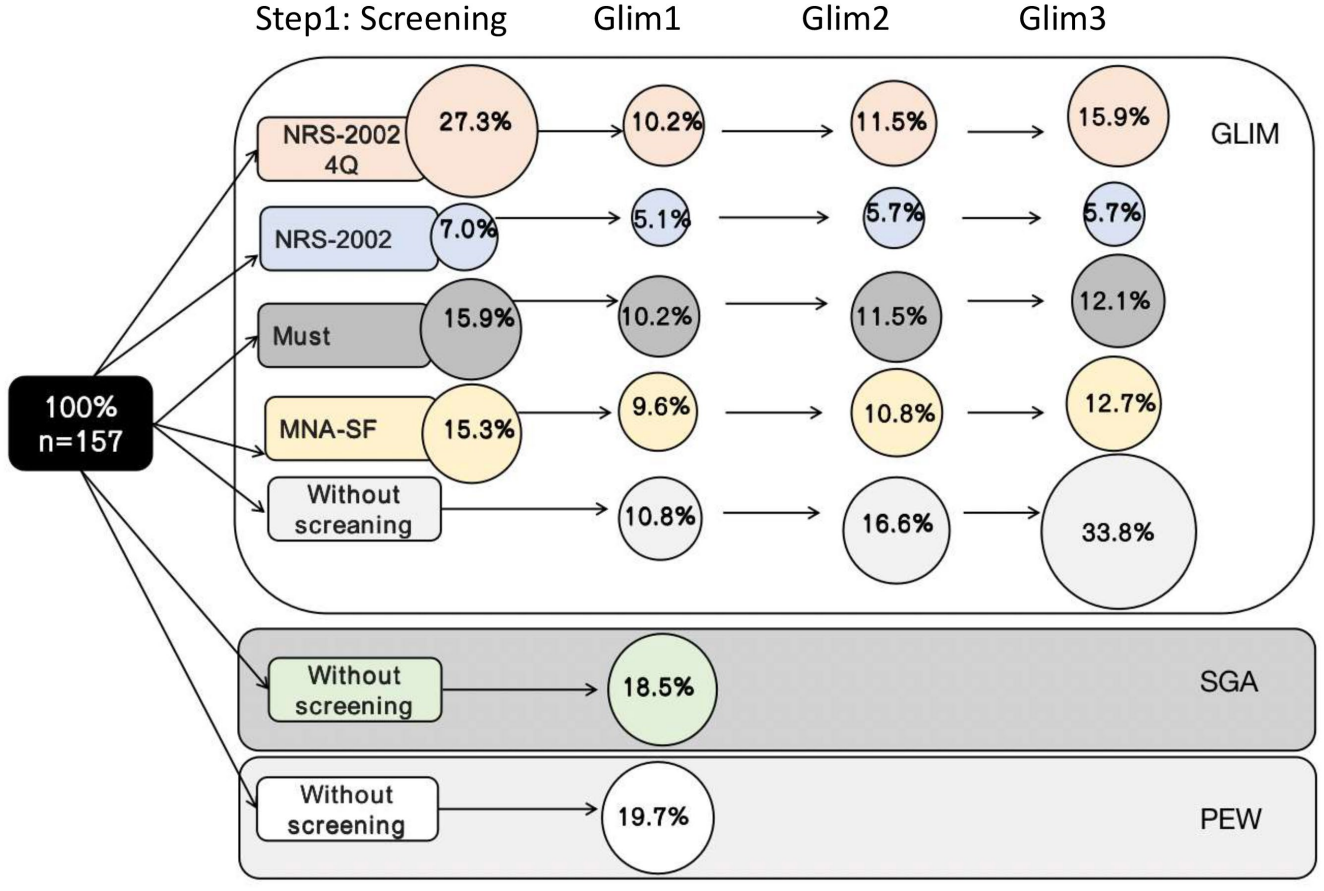
Results of the total GLIM process showing the number of patients diagnosed as malnourished when different screening tools were used in step 1 of the GLIM process.

The results of the entire GLIM process are presented in Table 2. The prevalence of malnutrition according to GLIM was different according to the nutritional risk screening tool AND to the method used to assess the low muscle mass. Depending on the screening tool, 7%–27.3% of patients were found to be at risk for malnutrition, and the NRS-2002-4Q identified more participants at risk for malnutrition than the other screening tools. The prevalence of malnutrition diagnosed in the GLIM was 10.2%–15.9% when screened with NRS-2002-4Q, 5.1%–5.7% with NRS-2002, 10.2%–12.1% with MUST, 9.6%–12.7% with MNA-SF, and 10.8%–33.8% without. The prevalence of malnutrition when applying different nutritional risk screening tools is shown in Figure 3.

**Table 2:** Results from the entire GLIM process, showing the number and proportion of patients diagnosed with malnutrition when different screening tools were used or not used in the GLIM process.

Step 1	Screening, *n* (%)	GLIM1, *n* (%)	GLIM2, *n* (%)	GLIM3, *n* (%)
NRS-2002-4Q	43 (27.3)	16 (10.2)	18 (11.5)	25 (15.9)
NRS-2002	11 (7.0)	8 (5.1)	9 (5.7)	9 (5.7)
MUST	25 (15.9)	16 (10.2)	18 (11.5)	19 (12.1)
MNA-SF	24 (15.3)	15 (9.6)	17 (10.8)	20 (12.7)
Without screening		17 (10.8)	26 (16.6)	53 (33.8)

### 3.3 Agreement between GLIM and SGA and PEW

Table 3 shows that the κ test revealed moderate agreement between the PEW and SGA (κ = 0.423, *p* < 0.001). Compared with SGA, PEW had a sensitivity of 55.2%, specificity of 88.3%, PPV of 51.6%, and NPV of 89.7% for the diagnosis of malnutrition.

**Table 3:** Assessment result of PEW and SGA.

PEW	SGA		Sum
	Malnutrition	Non-malnutrition	
PEW	16	15	31
Non-PEW	13	113	126
Sum	29	128	157

As shown in Table 4, a consistency test was conducted between GLIM and SGA. There was no statistical difference between SGA and GLIM2 when using the MUST, and when using the NRS-2002-4Q to screen for nutritional risk, GLIM1 and GLIM2 were in fair agreement with the SGA (GLIM1: κ = 0.258, 95% CI: 0.02–0.39, *p* = 0.001; GLIM2 κ = 0.331, 95% CI: 0.14–0.52, *p* < 0.001); GLIM3 was moderately consistent with SGA (κ = 0.464, 95% CI: 0.28–0.65, *p* < 0.001).

**Table 4:** Assessment result of GLIM and SGA.

Screening tool	SGA	GLIM	κ (95% CI)	*P*	Sensitivity%	Specificity%	PPV%	NPV%
NRS-2002 4Q	29	GLIM1	0.258 (0.07–0.45)	0.001	27.6	93.8	50.0	85.1
		GLIM2	0.331 (0.14–0.52)	<0.001	34.5	93.8	55.6	86.3
		GLIM3	0.464 (0.28–0.65)	<0.001	51.7	92.2	60.0	89.4
NRS-2002	29	GLIM1	0.207 (0.02–0.39)	0.001	17.2	97.7	62.5	83.9
		GLIM2	0.250 (0.06–0.44)	<0.001	20.7	97.7	66.7	84.5
		GLIM3	0.250 (0.06–0.44)	<0.001	20.7	97.7	66.7	84.5
MUST	29	GLIM1	0.207 (0.02–0.40)	0.006	24.1	93.0	43.8	84.4
		GLIM2	0.182 (0.00–0.37)	0.018	24.1	91.4	38.9	83.6
		GLIM3	0.219 (0.03–0.41)	0.05	27.6	91.4	42.1	84.2
MNA-SF	29	GLIM1	0.220 (0.03–0.41)	0.003	24.1	93.8	46.7	84.5
		GLIM2	0.232 (0.04–0.42)	0.003	27.6	92.2	47.1	84.9
		GLIM3	0.239 (0.06–0.42)	0.003	37.9	85.9	55	85.9
Without screening	29	GLIM1	0.245 (0.05–0.44)	0.001	27.6	93.0	47.1	85.0
		GLIM2	0.396 (0.20–0.59)	<0.001	37.9	95.3	42.3	87.1
		GLIM3	0.295 (0.14–0.45)	<0.001	65.5	73.4	35.8	90.4

As shown in Table 5, a consistency test was conducted between GLIM and PEW. The highest agreement between the GLIM and PEW was observed when using the NRS-2002 4Q as a nutritional risk screen. PEW was not statistically different from GLIM with NRS-2002; GLIM3 with MUST, and GLIM3 without screening.

**Table 5:** Assessment results of GLIM and PEW.

Screening tool	PEW	GLIM	κ (95% CI)	*P*	Sensitivity%	Specificity%	PPV%	NPV%
NRS-2002 4Q	31	GLIM1	0.189 (0.01–0.37)	0.011	22.6	92.9	43.8	83
		GLIM2	0.26 (0.07–0.45)	0.001	29	92.9	50	84.2
		GLIM3	0.306 (0.12–0.49)	<0.001	38.7	89.7	48	85.6
NRS-2002	31	GLIM1	0.044 (−0.10–0.19)	0.515	9.7	93.7	27.3	80.8
		GLIM2	0.074 (−0.16–0.16)	0.297	12.9	92.9	30.8	81.3
		GLIM3	0.074 (−0.16–0.16)	0.297	12.9	92.9	30.8	81.3
MUST	31	GLIM1	0.238 (0.05–0.42)	0.001	25.8	93.7	50	83.7
		GLIM2	0.188 (0.01–0.37)	0.015	25.8	90.5	40	83.2
		GLIM3	0.176 (−0.004–0.36)	0.023	25.8	89.7	38.1	83.1
MNA-SF	31	GLIM1	0.202 (0.02–0.38)	0.006	22.6	93.7	46.7	83.1
		GLIM2	0.2 (0.02–0.38)	0.009	25.8	91.3	42.1	83.3
		GLIM3	0.21 (0.03–0.39)	0.007	29	89.7	40.9	83.7
Without screening	31	GLIM1	0.225 (0.04–0.41)	0.003	25.8	92.9	47.1	83.6
		GLIM2	0.228 (0.05–0.41)	0.004	35.5	86.5	39.3	84.5
		GLIM3	0.13 (−0.15–0.15)	0.072	51.6	65.9	27.1	84.7

Regardless of the nutritional screening tool used, the sensitivity magnitude was as follows: GLIM3 ≥ GLIM2 ≥ GLIM1. The diagnostic criterion of RMM is crucial in GLIM, and the addition of HGS increases sensitivity. However, the sensitivity of the GLIM did not reach acceptable levels, regardless of the screening tool used. The specificity of all screening tools improved to acceptable levels compared with no screening. The NPV of all screening tools were acceptable.

## 4 Discussion

Malnutrition is a global problem, and its diagnosis remains a major challenge for healthcare organizations and non-nutrition specialists. The causes of malnutrition in chronic kidney disease are chronic inflammation, intestinal dysbiosis, metabolic acidosis, insulin resistance, infection, and oxidative stress (36). A total of 11%–54% of patients with stage 3–5 are malnourished worldwide (37). Moreover, morbidity positively correlates with chronic renal function staging (38). In the present study, the prevalence of malnutrition in patients with CKD was generally consistent with that reported in the literature.

### 4.1 Consistency between SGA, PEW, and GLIM

The SGA is an effective tool for the nutritional assessment of hospitalized patients and is often used as the gold standard in research studies (10, 20). PEW is used to characterize the loss of body proteins and energy reserves associated with kidney disease (11). The GLIM aims to standardize the diagnosis of malnutrition in the clinical setting. In our study, the consistency test of the SGA and GLIM was moderately consistent; the consistency between the GLIM and PEW was general consistency. The first step with nutritional risk screening showed acceptable specificity and NPV (>80%) and lower sensitivity and PPV between GLIM, SGA, and PEW. GLIM can better screen patients without malnutrition but lacks accuracy in screening malnourished patients, which may be related to lack of clarity in defining the program content of GLIM. In a study of two maintenance hemodialysis (MHD) patient cohorts, the GLIM showed low agreement in identifying malnourished participants using either a seven-point participative global assessment or a malnutrition inflammation score (39). This finding is consistent with our results.

The GLIM has been studied in other clinical settings, including cancer (40), inflammatory bowel disease (41) and healthy community-dwelling older adults (42). In a study of gastric cancer, the GLIM criteria were moderately consistent with Patient-generated Subjective Global Assessment (PG-SGA; κ = 0.548) (40). In a meta-diagnosis of malnutrition by GLIM, the accuracy of the GLIM criteria for the diagnosis of malnutrition was assessed (using a variety of validated nutritional assessment tools, including the SGA, as a reference standard), and according to the results of a subgroup analysis using the SGA as a reference standard, the GLIM criteria had a better diagnostic value (sensitivity 0.81; specificity 0.80), (43). This difference may be due to differences in study populations. Patients in these studies were at a higher risk of malnutrition and had more severe disease.

### 4.2 Impact of screening tools

We used four nutritional screening tools to determine the risk of malnutrition in patients with CKD. Depending on the screening tool, the percentage of patients at risk of malnutrition ranges from 7 to 27.3%. The NRS-2002-4Q identified more participants at risk of malnutrition than the other screening tools. However, it was not possible to identify many patients with malnourishment. In a study of colorectal cancer (CRC) patients study, nutritional risk was assessed using the NRS-2002-4Q, MUST, Malnutrition Screening Tool (MST), and PG-SGA to assess nutritional risk and subsequently assess the concordance of the GLIM with the PG-SGA. The results showed that the PG-SGA short form (PG-SGA-SF) and NRS-2002-4Q were the most appropriate screening tools (26).

However, a mixed-population study comparing the concordance of the GLIM with PG-SGA showed that the GLIM without the NRS-2002 as a nutritional risk screen was more concordant than the NRS-2002 (44). This difference may be due to the different age groups of the study population and the study population. The prevalence of malnutrition based on the GLIM process varied depending on the choice of the nutritional screening tool. To directly compare malnutrition rates across studies, the GLIM criteria must be further standardized.

### 4.3 RMM

The RMM has been omitted from some studies because of racial and sex differences, discrepancies in measurements, and the absence of accurate threshold references (20, 21). Many humans become overweight and obese, and a low BMI does not accurately reflect malnutrition. RMM should be evaluated at all times for the phenotypic criterion in patients with CKD (19, 45, 46). In our study, the addition of HGS increased the sensitivity. To date, different studies have used different methods and reference values to measure muscle loss. This lack of criteria has led to uncertainty in the clinical application of the GLIM criteria. It is expected that more studies will be conducted in the future to determine the definitions of the criteria for different sexes, ages, and ethnic groups.

### 4.4 Implications

First, different screening tools were carefully selected as step 1 of the GLIM process because of their impact on the prevalence of malnutrition and their consistency with reference methods. Clinicians should indicate which tool is used to screen for the risk of malnutrition when reporting the prevalence of malnutrition in the GLIM. Second, the RMM is integral to the GLIM diagnostic process. The metrics of RMM coupled with HGS will increase the sensitivity of the GLIM in detecting insidious muscle mass loss in more patients. To compare the prevalence of malnutrition in different studies and reach a comprehensive consensus on the diagnosis of malnutrition, the existing GLIM process must be improved or standardized.

### 4.5 Strengths and limitations

The strength of this study is that it is the first to validate the applicability of the GLIM in a population of non-dialysis CKD patients using the SGA as the gold standard. The applicability of PEW and GLIM was also validated. As recommended by the GLIM validation guidelines, the reliability of the GLIM criteria was first validated using several commonly used nutritional screening tools. In addition, the use of bioelectrical impedance measures, SMI and FFMI, to represent the RMM in the GLIM process was more accurate. Muscle mass is an indispensable phenotypic criterion and HGS increases the sensitivity of GLIM.

This study also has some limitations. First, this was a single-center study with a relatively limited study population, and a multicenter study should be conducted. Second, the sample size should be enlarged to reduce sample bias and make the results more reliable.

## 5 Conclusion

The PEW showed moderate agreement with the SGA in the diagnosis of malnutrition. The agreement of the GLIM with the SGA and PEW was not acceptable, but the agreement of the initial screening using the NRS-2002 4Q was better. The addition of HGS increased sensitivity. The consistency of the GLIM with the SGA and PEW depends on the nutritional screening tool used and the method used to assess the RMM. In future studies, a consensus is required on how to implement the GLIM process, and the existing GLIM needs to be improved.

## Data availability statement

The original contributions presented in the study are included in the article/Supplementary material, further inquiries can be directed to the corresponding authors.

## Ethics statement

This study was conducted in accordance with the Declaration of Helsinki and was approved by the Ethics Committee of the Chinese People’s Liberation Army General Hospital (approval number S2022-324-01). All participants provided signed informed consent and agreed to participate in the study. The studies were conducted in accordance with the local legislation and institutional requirements.

## Author contributions

HH: Data curation, Investigation, Writing – original draft. QW: Conceptualization, Writing – original draft. YLu: Writing – original draft. ZT: Methodology, Writing – original draft. FL: Formal Analysis, Software, Writing – original draft. RZ: Formal Analysis, Writing – original draft. GC: Resources, Writing – review & editing. JH: Resources, Visualization, Writing – review & editing. LZh: Resources, Supervision, Writing – review & editing. LZe: Formal Analysis, Project administration, Writing – review & editing. XCa: Resources, Supervision, Writing – review & editing. JY: Formal Analysis, Project administration, Writing – review & editing. YW: Resources, Supervision, Writing – review & editing. KW: Formal Analysis, Software, Writing – review & editing. YLi: Formal Analysis, Methodology, Writing – review & editing. QL: Software, Writing – review & editing. XCh: Writing – review & editing, Resources. ZD: Conceptualization, Writing – review & editing, Resources.
