# Supplementary material for: Validity and applicability of the global leadership initiative on malnutrition criteria in non-dialysis patients with chronic kidney disease
Source: Front Nutr. 2024 Feb 1;11:1340153. doi: 10.3389/fnut.2024.1340153 (PMC10867223; doi:10.3389/fnut.2024.1340153)
Supplement: Supplementary file 1 [file Table_1.DOCX]

Supplementary Material

Validity and Applicability of the Global Leadership Initiative on Malnutrition (GLIM) Criteria in Non-dialysis Patients with Chronic Kidney Disease

Hui Huang1,2 †，Qian Wang1†，Yayong Luo1,2，Zhengchun Tang1,2，Fang Liu1,2，Ruimin Zhang1,3，Guangyan Cai1，Jing Huang1，Li Zhang1，Li Zeng1，Xueying Cao1，Jian Yang1，Yong Wang1，Keyun Wang1 ，Yaqing Li1 ，Qihu Li1,2，Xiangmei Chen1,2*，Zheyi Dong1*

*** Correspondence:** Corresponding Author: Xiangmei Chen :xmchen301@126.com ；Zheyi Dong:shengdai26@163.com

# Supplementary Tables

Table 1. Nutritional status assessment of the study population.

| Assessment Tool | Item | Total (n, %) | Male (n, %) | Female (n, %) | p Value |
| --- | --- | --- | --- | --- | --- |
| SGA | Weight change | 11（7%） | 6（6.2%） | 5（8.3%） | 0.849 |
|  | Dietary intake change | 19（12.1%） | 14（ 14.4%） | 5（8.3%） | 0.255 |
|  | Gastrointestinal symptoms | 12（7.6%） | 6（6.2%） | 6（10%） | 0.572 |
|  | Functional capacity | 64（40.8 %） | 36（37.1 %） | 28（46.7 %） | 0.237 |
|  | Metabolic demand | 157（100%） | 97（100%） | 60（100%） | - |
|  | Subcutaneous fat loss | 58（36.9%） | 35（36.1%） | 23（38.3%） | 0.776 |
|  | Muscle wasting | 26（16.6%） | 17（17.5 %） | 9（ 15%） | 0.679 |
|  | Edema | 36（22.9%） | 21（ 21.6%） | 15（ 25%） | 0.627 |
|  | Result of SGA | 29（18.5 %） | 16（ 16.5%） | 13（21.7%） | 0.417 |
| PEW | Serum chemistry | 92（58.6%） | 52（ 53.6%） | 40（66.7%） | 0.106 |
|  | Body mass | 53（33.8%） | 25（25.8%） | 28（46.7%） | 0.007 |
|  | Muscle mass | 16（10.2%） | 10（ 10.3%） | 6（10%） | 0.950 |
|  | Dietary intake | 116（73.9%） | 68（70.1%） | 48（80%） | 0.170 |
|  | Result of PEW | 31（19.7%） | 16（16.5%） | 15（25%） | 0.193 |
